# Combined training is not superior to strength and aerobic training to mitigate cardiovascular risk in adult healthy men

**DOI:** 10.5114/biolsport.2022.107483

**Published:** 2021-08-30

**Authors:** Reginaldo Gonçalves, Daisy Motta-Santos, Leszek Szmuchrowski, Bruno Couto, Ytalo M. Soares, Vinícius de O. Damasceno, Gustavo F. Pedrosa, Marcos D. M. Drummond, Fernando V. Lima, Alexandre S. Silva

**Affiliations:** 1 Sports Department, Federal University of Minas Gerais, Belo Horizonte, Brazil; 2 University of the Sunshine Coast, Queensland, Australia; 3 Physical Education Department, Federal University of Paraiba, João Pessoa, Brazil; 4 Physical Education Department, Federal University of Pernambuco, Recife, Brazil

**Keywords:** Combined training, Health, Cardiovascular risk, HDL-C, Aerobic training

## Abstract

Although the beneficial effects of aerobic training on cardiovascular risk factors are evident, the potential beneficial effect of strength and combined training on these risk factors is controversial. This study aimed to evaluate the effect of aerobic and strength training programmes, performed alone or in combination, on cardiovascular risk factors in sedentary, apparently healthy and non-obese adult men. The study was conducted with 37 subjects who were randomly divided into the following groups: aerobic (AG), combined (ASG), strength (SG) and control (CG). The exercise programmes were performed three times a week and lasted approximately 50 minutes. Dietary intake, anthropometry, blood pressure, muscular strength, aerobic capacity, lipid profile and glycaemic control were assessed before and after 12 weeks of the intervention. One-way analysis of variation (ANOVA) for baseline, and ANOVA for repeated measures were used to assess differences between the initial and final time points of the four groups. Changes in blood pressure and glycaemic control were not significant in any of the groups. No differences were observed in LDL-C between training groups. HDL-C increased significantly only in the AG. In conclusion, if minimal changes in the lipid profile are needed, an aerobic training programme can provide possible benefits for HDL-C in apparently healthy and non-obese adult men.

## INTRODUCTION

Different types of exercise can bring benefits and reduce some cardiovascular risk factors [1, 2]. Over the last 20 years, the literature has revealed that aerobic training programmes that expend at least 1000 kcal/week may promote reductions in serum concentrations of triglycerides and increase high-density lipoprotein (HDL–C), particularly HDL2-C [3–7]. Recent studies indicate that low-densitylipoprotein (LDL-C) is also reduced in response to aerobic training [8] in people with hyperlipidaemia. A significant reduction in glycaemia and an improvement in insulin sensitivity occur in diabetic people who perform aerobic training by improving the activity of GLUT-4 and enzymes related to the intracellular cascade in which glucose is captured at the muscle [9]. Likewise, it is well established that aerobic training programmes are effective in promoting reductions in systolic and diastolic blood pressure [4].

A small number of studies have demonstrated the benefit of strength training programmes [6, 10] but there are sufficient reviews and meta-analyses that indicate reductions in LDL-C and total cholesterol as well as increase of HDL [11, 12] in addition to reduction of the HOMA-IR index. Strength training programmes promote more discreet reductions in cardiovascular risk factors. A review by Fagard [4] described a significant decrease in diastolic blood pressure of 3.5 mmHg (p < 0.01) but a non-significant decrease in systolic blood pressure of 3.2 mmHg (p = 0.10).

Recently, researchers have shown increasing interest in investigating the combination of aerobic and strength exercise programmes, which may result in additional effects on cardiovascular risk factors [13–17]. Data from a systematic review indicated a significant lowering of systolic blood pressure after combined exercise training (-3.59 mmHg, 95% CI, -6.93 to -0.24) [18]. Additionally, this training protocol (combined) caused a significant reduction in glucose in women with metabolic syndrome; however, there is no body of evidence that confirms these previous data. Data that have verified the effect of combined training on insulin sensitivity are even scarcer.

This current perspective indicates that although the benefits of aerobic training for cardiometabolic status are evident, there is still a need for studies to construct a body of evidence regarding the potential beneficial effect of strength training, primarily from the combination of aerobic and strength training, on cardiometabolic parameters. Additionally, the available studies have only investigated populations that already have cardiometabolic dysfunction. Adults who are considered to have normal parameters could benefit, from a preventive perspective, if the physical training promotes improvement in these indicators. However, previous studies, especially those investigating strength and combined training, are not sufficient to determine the potential of physical training in improving these cardiometabolic indicators in this population.

Therefore, the objective of this study was to evaluate the effect of aerobic and strength training programmes, performed alone or in combination, on cardiovascular risk factors (blood pressure, lipid and glycaemic status) in sedentary adult men who are apparently healthy, eutrophic or overweight.

## MATERIALS AND METHODS

### Experimental Design

A randomized intervention was conducted for 12 weeks. The volunteers performed initial assessments of dietary intake, anthropometry, blood pressure, muscular strength, and aerobic capacity and provided blood samples to assess lipid levels (total cholesterol, HDL-C, LDL-C) and glycaemic control (glucose and insulin). Subsequently, the experimental groups started their training protocols that lasted 12 weeks. At 24 to 48 hours after the last training session, the experimental groups and control group (CG) repeated the initial tests.

### Subjects

The volunteers recruited for the study were male, aged between 30 and 60 years, who were not at increased risk for cardiovascular disease (circumference of waist less than 102 cm and BMI below 30 kg/m^2^); performed less than 600 METs per week in physical activities (according to the International Physical Activity Questionnaire – IPAQ); and were able to practise regular physical activity (according to the Physical Activity Readiness Questionnaire – PAR-Q).

The selection of volunteers for the study took place after extensive internal advertising on the university campus (internet, radio, TV and posters). Forty-six apparently healthy and previously sedentary adult males were able to participate in the study considering inclusion criteria. The subjects were distributed according a table of random numbers generated by Excel (Microsoft Office for Windows, 2010, Microsoft Corp. WA, USA) into four groups: the aerobic training group (AG) (n = 12), the strength training group (SG) (n = 12), the aerobic and strength training group (ASG) (n = 12) and a control group (CG) in which no exercise was performed (n = 10).

The inclusion criteria were: did not use medications including statins, beta-blockers or hypoglycaemic agents; no smoking history; no endocrine or immune abnormalities; heart, mental or thyroid disease, no diabetes or hypertension. We did not include in the study subjects who were in controlled dietary intervention. Volunteers were disqualified if they did not perform at least 75% of the training sessions (28 sessions), missed three consecutive sessions, did not perform the initial or final evaluation tests, or experienced some sort of injury during the study period. The participants completed an informed consent document, which outlined the experiment and was previously approved by the Ethics Committee of Federal University of Minas Gerais (Protocol 264-755-2013). At the end of the intervention, we gave the subjects in the CG the possibility of choosing any type of training intervention (aerobic, strength or combined) used in the study.

### Procedures

#### Nutritional directions

A two-day non-consecutive food record was provided. Nutritional behaviour was analysed using the nutritional software Dietpro5i (Viçosa, Brazil) [19]. The volunteers were instructed to maintain their eating habits throughout the entire study. The food record was collected at the end of the intervention.

#### Assessment of Aerobic Capacity and Strength

Aerobic capacity was assessed using the submaximal Astrand ergometer test [20]. The 4–6 maximum repetitions test proposed by Dohoney et al. [21], involving bench press and knee extension exercises, was used to estimate muscle strength of the upper and lower limbs through specific equations for weight prediction of one maximum repetition for each exercise. For comparison, muscle strength was assessed relative to the total body mass of the individual.

#### Blood pressure and resting heart rate

These parameters were measured according to the recommendations of the Brazilian Society of Cardiology using the automatic device Omron HEM-7200 (Omron Healthcare Co. Ltd, China), which has been validated for scientific research [22]. The arm circumference was previously measured if necessary, to fit the cuff size. The blood pressure measurements were performed on the right arm of the volunteers. The same device was used to measure and record the resting heart rate.

#### Blood collection and biochemical analyses

After 12 hours of fasting, blood samples were withdrawn from a peripheral vein and placed in anticoagulant tubes for analysis of glucose (2.5 mL) and in dry tubes for further analysis (7.5 mL). Blood samples were centrifuged at 3,000 rpm for 10 minutes. Total cholesterol, LDL-C, HDL-C and glucose were measured using a spectrophotometric automatic colorimetric enzymatic reaction in the Bioclin Kit auto analyser (Wiener lab – Brazil). Insulin was analysed from 1 mL of serum using an electrochemiluminescence method. Data on insulin and glucose were used to determine insulin sensitivity according to the HOMA-IR protocol (Homeostasis Model Assessment Insulin Resistance Index) [23]. The protocol uses the following equation: ([fasting serum insulin (*µ*U/mL) × fasting plasma glucose (mmol/L)] / 22.5).

### Training Protocols

The experimental groups performed three training sessions per week, lasting 50 minutes each for 12 weeks, each lasting 36 sessions. The training exercise protocols were conducted in the Physical Education School at the campus of Federal University of Minas Gerais and all sessions were monitored by researchers and trainees.

### Aerobic Training Group (AG)

Aerobic training was performed on a treadmill and stationary bike in a randomized order, and the order of the equipment was alternated within the same session. The training intensity was monitored using a heart rate (HR) monitor (Polar FT1, Polar Electro, Kempele, Finland). The heart rate reserve (HRreserve) was calculated for each individual (HRreserve = maximum HR – resting HR). The maximum heart rate (MHR) was estimated with the equation 220bpm – age (years). The desired percentage of the HR reserve was then added to the resting HR value to determine the HR training. Volunteers were encouraged to remain at the prescribed HR training level, but fluctuations were allowed up to approximately ± 10 bpm. The progression of moderate intensity aerobic training based on the guidelines of the American College of Sports Medicine (ACSM) [24] is described in Table 1.

**TABLE 1:** Training Plan

Weeks	Aerobic Training load	Weeks	Strength Training load
1	30 min – 50% HR reserve	1	1 series of 8 to 12 RM in 10 exercises
2	35 min – 50% HR reserve	2	2 series of 8 to 12 RM in 10 exercises
3	40 min – 50% HR reserve	3–5–7–9–11	3 series of 8 to 12 RM in 10 exercises
4	45 min – 50% HR reserve		
5–8	50 min – 55% HR reserve	4–6–8–10–12	3 series of 4 to 6 RM in 10 exercises
9–12	50 min – 60% HR reserve		

HR reserve: heart rate reserve, RM: repetition maximum.

### Strength Training Group (SG)

Strength training was performed using the following strength exercises: horizontal leg press, knee extension machine, bench press, seated knee flexion machine, Smith bench press, lat pull-down machine, seated rowing machine, dumbbell shoulder abduction, dumbbell arm curl, pull down triceps, abdominal crunch and trunk extension machine. At the beginning of each training session, five minutes of fast walking (± 6 km/h) and five minutes of general stretches were performed. The strength training progression is depicted in Table 1. In the strength training exercise, for each exercise, the duration of each repetition was approximately three seconds, and one-minute intervals were given between each series and between each exercise. All training sessions were individually monitored by the research team. When volunteers reached the higher limit of repetitions in the set, the weight was increased and the number of repetitions remained close to the lower limit prescribed for the series (e.g., 8–12). The total duration of each strength training session was approximately 50 minutes, and the training load was based on ACSM guidelines [24].

### Aerobic and Strength Training Group (ASG)

The combined ASG was always performed by alternating strength and aerobic training in the same session. The order of exercises in the training sessions (strength and aerobic) was alternated each week. As the two types of training were performed within the same session, the length of aerobic training and the total number of series in strength training were reduced by half. The strength training was conducted similarly to SG, but with half of the stimulus volume. To perform half of the total volume in relation to SG, from week 3, five exercises were performed with 2 sets (knee extension machine, knee flexion machine, Smith bench press, lat pull-down, abdominal crunch), and 5 exercises were performed with one set (horizontal leg press, dumbbell shoulder abduction, dumbbell arm curl, pull down triceps, trunk extension machine).

### Control Group (CG)

The control group performed all procedures before and after 12 weeks including nutritional directions, assessment of aerobic capacity and strength, blood pressure, resting heart rate measurements and blood collection for biochemical analyses.

### Statistical Analyses

Data are presented as the mean and standard error of the mean. The Shapiro-Wilk and Levene tests were initially used to test the normality and homogeneity of the data. To compare the initial conditions of the four groups, the one-way ANOVA test was used, and the ANOVA for repeated measures was used to assess differences between the initial and final statuses of the four groups.

## RESULTS

At baseline, the volunteers in the four groups had similar ages and BMI classification between normal and overweight but the body composition variables (fat and muscle mass) were similar between the groups (Table 2). Aerobic capacity was rated as “good” in the four groups according to the ACSM [24], and there were no significant differences. There was a sample loss of 20% for nutritional variables, but these data indicated that the groups had similar total caloric intake of total calories, carbohydrates, lipids and proteins, as well as intake of food sources of cholesterol. There were no noticeable changes between pre- and post-intervention nutritional intake (total calorie intake: AG: 2,050.9 ± 484.4 versus 2,133.6 ± 810.0 kcal; SG: 2,032.0 ± 533.2 versus 2,163.9 ± 547.2 kcal; ASG: 2,170.9 ± 639.9 to 2,139.0 ± 521.1 kcal and CG: 2,202.4 ± 771.4 versus 1,914.6 ± 576.4 kcal).

**TABLE 2:** Data at Baseline

	CG(n = 8)	AG(n = 11)	SG(n = 8)	ASG(n = 10)
Age (years)	41.8 ± 2	39.3 ± 3	37.8 ± 3	38.8 ± 2
Weight (Kg)	75.5 ± 3	76.0 ± 3	76.8 ± 3	79.6 ± 2
BMI (kg/m^2^)	25.4 ± 0.8	25.7 ± 1	24.5 ± 1	25.1 ± 0.8
Waist girth (cm)	87.4 ± 3	88.1 ± 2	88.9 ± 3	88.7 ± 2
Sum of 4 SF (mm)	73.5 ± 11	80.2 ± 6	78.8 ± 11	71.5 ± 6
SBP (mmHg)	122 ± 5	120.6 ± 4	119.8 ± 2	121.0 ± 2
DBP (mmHg)	78.6 ± 4	72.9 ± 3	73.0 ± 2	70.7 ± 2
VO2max (ml·kg^-1^min^-1^)	37.7 ± 2	39.9 ± 3	38.3 ± 3	42.6 ± 3
TC (mg/dL)	187.2 ± 10	209.3 ± 14	186.7 ± 17	225.0 ± 10
LDL-C (mg/dL)	115.2 ± 10	138.1 ± 13	116.8 ± 13	136.8 ± 8
HDL-C (mg/dL)	45.4 ± 4	46.4 ± 2	40.5 ± 5	50.7 ± 3
Triglycerides (mg/dL)	108.8 ± 20	124.2 ± 20	99.2 ± 11	187.5 ± 45
Glucose (mg/dL)	95.8 ± 3	95.9 ± 2	89.9 ± 3	88.8 ± 2
Insulin (*μ*U/L)	8.7 ± 1.2	7.9 ± 0.9	8.3 ± 1.7	5.6 ± 0.7
HOMA	37.0 ± 5	34.0 ± 4	33.8 ± 7	22.4 ± 3

Data are presented as means and standard deviations. CG = Control Group; AG = Aerobic Training Group; SG = Strength Training Group; ASG = Aerobic and Strength Training Group; BMI = Body Mass Index; SF = Skinfold; SBP = Systolic Blood Pressure; DBP = Diastolic Blood Pressure; VO_2_max = Maximal Aerobic Capacity; TC = Total Cholesterol; LDL-C = Low Density Lipoproteins; HDL-C = High Density Lipoproteins;HOMA = Homeostasis Model Assessment.

All groups had blood pressure, glucose, insulin and HOMA values within normal limits before intervention, according to the criteria of the Brazilian Society of Cardiology [25] and the Brazilian Society of Diabetes [26]. Although the ASG had glucose and HOMA levels approximately 35% lower than those of the other groups, there were no significant differences in these variables between the groups.

### Cardiovascular Risk Parameters

**FIG. 1:**
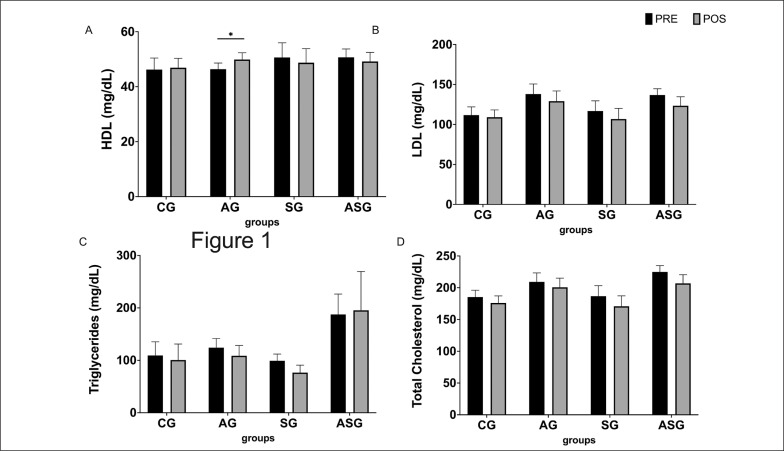
Effect of aerobic training protocols, strength or combined (aerobic + strength) on the lipid profile HDL-C (a) and triglycerides (b) of adults. AG = aerobic training group; SG = strength training group; ASG = aerobic and strength training group; CG = control group. *intra-group difference (pre- vs. post-test), p < 0.05.

Regarding total cholesterol, the AG, SG and ASG demonstrated reductions of 8.5 ± 5 mg/dL, 15.9 ± 9 mg/dL and 18.1 ± 9 mg/dL, respectively, whereas the level increased by 7.1 ± 17 mg/dL in the CG. However, these changes were not significant in either group. Despite the lack of significance in reducing total cholesterol, groups that performed the aerobic and combined training had values above the normal limit before starting the training programme and completed the study with almost normal values (200.14 ± 7 mg/dL and 206.9 ± 14 mg/dL, respectively) (Figure 1, panel A).

The LDL-C changes were similar to those observed with TC, with a greater difference in the group that performed combined training (-23.6 ± 14 mg/dL) compared to the groups that performed aerobic training (-8.9 ± 5 mg/dL) and strength training (-10.1 ± 9 mg/dL). There was no significant difference between these values, and no differences between pre- and post-training in any of these groups were found. The control group showed a more discreet reduction, only -2.8 ± 10 mg/dL (Figure 1, panel B).

The AG was the only programme able to promote a significant change in any component of the lipid profile (an increase of + 3.5 ± 1.5 mg/dL in HDL-C). The SG and ASG showed a slight but not significant reduction in HDL and the CG showed no changes (Figure 1, panel C). There were no significant changes in triglycerides in any group (Figure 1, panel D).

**FIG. 2:**
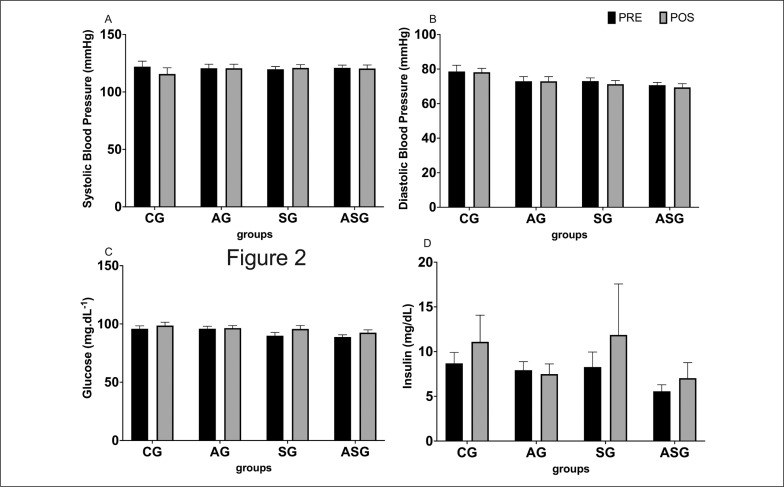
Effect of aerobic training protocols, strength or combined (aerobic + strength) on blood pressure (a, b), glycaemia and insulin profile (c, d). AG = aerobic training group; SG = strength training group; ASG = aerobic and strength training group; CG = control group.

None of the training protocols evaluated caused any significant change in systolic or diastolic blood pressure (Figure 2, panels A and B).

Figure 2 (Panel C) shows that the exercise interventions promoted insignificant increases in blood glucose levels between + 0.5 mg/dL and + 5.8 mg/dL, but the final values did not exceed the normal limits established by the Brazilian Society of Endocrinology and Metabolism [26]. None of the training protocols evaluated caused any significant change in insulin (Figure 2, panel D). As a result, the HOMA index remained almost unchanged between pre- and post-intervention training (33.9 ± 4.2 versus 32.9 ± 5.7 to AG; 33.7 ± 7.0 versus 27.1 ± 6.2 to SG; 23.8 ± 3.1 to 21.9 ± 2.4 to ASG; 37.3 ± 6.2 versus 36.6 ± 5.5 to CG).

### Aerobic Capacity Responses

**FIG. 3:**
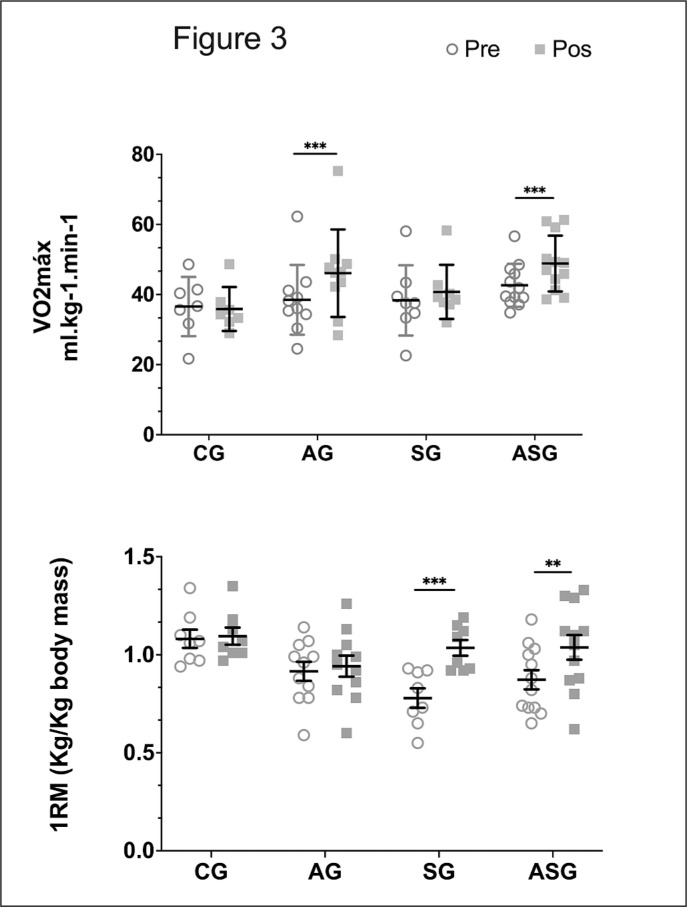
Effect of aerobic training protocols, strength or combined (aerobic + strength) on VO2max (above) and 1RM knee extension (below). AG = aerobic training group; SG = strength training group; ASG = aerobic and strength training group; CG = control group.

The AG and ASG demonstrated a significant increase in aerobic capacity of + 6.5 ± 5 ml·kg^-1^min^-1^ and + 6.2 ± 5 ml·kg^-1^min^-1^, respectively, without differences between the two groups (Figure 3). The experimental groups that did not perform aerobic training showed no significant difference in aerobic capacity between pre- and postintervention training.

### Strength Responses

The strength gain was evaluated through the strength exercise weight (kg) corrected by body mass (kg). Only the SG and ASG groups showed a significant increase in strength. The SG showed increases of 0.3 ± 0.08 kg/kg and 0.1 ± 0.07 kg/kg in load weight with the Smith bench press and knee extension machine, respectively. The ASG increased strength in these two exercises (0.2 ± 0.09 and 0.1 ± 0.09 kg/kg of body weight, respectively). The AG and CG showed no significant gains of strength between the beginning and the end of the study (Figure 3).

## DISCUSSION

Data from our study showed that HDL-C changed significantly in apparently healthy adults with a significant increase only in the AG. The ASG did not provide additional benefits in any of the variables investigated, although there was a distinct advantage of this type of training in reducing total cholesterol and LDL-C compared to aerobic and strength training performed in isolation. This is the first randomized study to compare the effects of aerobic, strength and combined training with similar training loads aiming to analyse the traditional cardiovascular risk factors in apparently healthy, non-obese men.

The primary influence of aerobic training on HDL-C has been described in previous studies [3, 6], although a study of 8 months of training only found a difference in triglycerides in the aerobic and combined groups [6]. There was a consensus on the effect of aerobic training on HDL-C until recently, as some studies have begun to show that this training protocol could also reduce LDL-C [8]. Based on this evidence, the data from this study reinforce the previous concept that only the HDL-C level is sensitive to aerobic training programmes, at least in healthy adults. The present data do not completely rule out the most recent indications that aerobic training can be beneficial in reducing LDL-C and total cholesterol [3, 16, 17]. Despite the absence of significant differences, participants in the aerobic and combined training groups, who began the study with total cholesterol levels above the normal range, benefited from the training once the desirable levels of this variable were almost reached. The descriptive results of the three forms of training (reductions between 8.5 and 18 mg/dL in total cholesterol) are within the ranges that have been presented in reviews, which support the effects of physical training on lowering total cholesterol [6, 12, 16]. Additionally, it should be emphasized that despite the lack of significant difference in reducing total cholesterol, the levels in the combined training group were approximately 11.3% and 14% higher than the levels in the aerobic and strength groups, respectively. The same tendency continued in relation to LDL-C, because the reduction promoted by the combined training was 165% and 133% higher than that obtained in the aerobic and strength training groups, respectively. Therefore, we recommend caution with respect to these two variables until studies with larger sample sizes and of longer duration are conducted.

The absence of significant changes in blood pressure are probably due to the initial values that were considered optimal values (approximately 120 mmHg for systolic pressure and below 80 mmHg for diastolic blood pressure), according to the literature [13, 15, 16, 27]. There is also agreement that the initial value of blood pressure is the variable that influences the effects of physical training on blood pressure the most, and fewer effects are expected from physical training as the blood pressure is closer to the ideal values [4].

In the study by Bateman and colleagues [28], only the combined group had a significant decrease in diastolic pressure. However, the training protocol of the combined group was created by adding the training volume of the aerobic and strength training protocols. In this study, the total training load was not equalized precisely across the groups, although a recent study that equalized the training loads showed substantial similarity to the loads of our training protocols [15].

The data related to glycaemic levels indicate the absence of any effect of training on this variable. Prior studies [11, 18] were conducted with people who had initial impairment of the glycaemic profiles, and this study does not change the state of knowledge on this issue. However, it serves to document the training effect in healthy, non-obese adults. Similar to blood pressure, decreases in glucose below the normal range could be harmful, so the body appears to modulate the effects of physical training within healthy limits.

Of the variables analysed (blood pressure, glucose and lipid profiles), the only variable that significantly changed was a variable in which the subjects had previous values different from the values considered ideal or optimal, and this change only occurred with physical training. This finding reinforces the notion that baseline cardiovascular risk factors appear to be the most important variable in the effects of training, rather than the presence of outcomes caused by these risk factors and the age of the participants or even the previous level of physical activity. It also seems to indicate that training has an effect only on variables that are already abnormal. The main practical implication of this study is that if minimal changes in the lipid profile are needed, an aerobic training programme is able to provide significant benefits for HDL-C and can promote benefits for total cholesterol and LDL-C, with the possibility of even more benefit with combined training instead of aerobic and strength training in isolation. Some possible methodological limitations of this study are the sample size and the short duration of intervention.

## CONCLUSIONS

Our study showed that HDL-C changed significantly in apparently healthy adults with a significant increase only in the aerobic exercise group (AG). The combined training (ASG) did not provide additional benefits in any of the cardiovascular risk factors investigated. In healthy volunteers who have cardiovascular risk biomarker values within the normal range, the training protocols performed do not show additional benefits despite having promoted improvements in aerobic capacity or strength. A physically active lifestyle could prevent unwanted changes in cardiovascular risk factors.

## Conflict of interest

The authors declare no conflict of interest.
